# Femtosecond X-rays illuminate disordered states during the early stages of crystallization

**DOI:** 10.1107/S2052252525005706

**Published:** 2025-07-01

**Authors:** Akinobu Niozu

**Affiliations:** a Graduate School of Humanities and Social Sciences Hiroshima University Higashi-Hiroshima 739-8524 Japan

**Keywords:** crystallization, X-ray free-electron lasers, XFELs, correlated fluctuations, time-resolved studies, nanocrystals

## Abstract

Crystallization is a fundamental non-equilibrium process in materials science, yet its early transient states remain difficult to probe experimentally. Möller *et al.* [(2025). *IUCrJ***12**, 462–471] use femtosecond X-ray scattering and X-ray cross-correlation analysis to reveal the structural evolution of defect-containing crystals forming in a supercooled noble-gas liquid.

Crystallization is a non-equilibrium process involving the formation and evolution of order from disorder. While much progress has been made in understanding stable crystal structures, the transient intermediate states that arise during the early stages of crystallization – often containing structural defects – remain elusive (Lupi *et al.*, 2017 ▸; Niozu *et al.*, 2021 ▸; De Yoreo, 2022 ▸). These defect-containing structures may play a pivotal role in determining both the nucleation rate (Lupi *et al.*, 2017 ▸; Möller *et al.*, 2024 ▸) and the final crystal morphology, yet our current understanding of them remains limited due to their fleeting nature.

There are several technical challenges associated with *in situ* investigations of crystallization processes. Crystalline defects can be probed by electron microscopy; however, such measurements are typically *ex situ* and cannot be easily extended to statistical investigations of large ensembles. Powder X-ray diffraction can be used for *in situ* investigations of the crystallization process on large ensembles, but it usually provides a one-dimensional (1D) intensity profile that corresponds to the orientational average of the three-dimensional (3D) intensity in reciprocal space. Crystalline defects can cause peak broadening in the 1D line profile; however, it is often challenging to distinguish between different types of defects solely based on the 1D information.

In the current issue of *IUCrJ*, Möller and co-workers report on the structures of disordered crystals forming in a supercooled noble-gas liquid (Möller *et al.*, 2025 ▸). Using an advanced scattering technique that combines femtosecond X-ray diffraction and X-ray cross-correlation analysis (XCCA) (Wochner *et al.*, 2009 ▸; Altarelli *et al.*, 2010 ▸; Kirian, 2012 ▸), they accessed the 3D reciprocal space of the disordered crystals and uncovered the structural evolution. They identified a decrease in stacking-fault occurrences in face-centered-cubic structures along the liquid jet – an insight that would be difficult to extract from the 1D powder pattern.

To understand how the XCCA technique provides such detailed structural insights, we briefly outline its fundamental principles, as illustrated in Fig. 1 ▸. When a femtosecond X-ray pulse illuminates a crystal, the resulting diffraction pattern captures only a two-dimensional (2D) slice of the 3D intensity distribution in reciprocal space, constrained by the geometry of the Ewald sphere. Reconstructing the full 3D information from such 2D slices is non-trivial without prior knowledge of crystal orientations. A common initial analysis is to derive the radial intensity profile of a diffraction image accumulated over many X-ray shots, yielding the familiar 1D powder diffraction pattern. To go beyond this 1D representation, one can analyze the correlations in the azimuthal intensity distribution of single-shot images. These angular correlations encode intrinsic features of the 3D intensity distribution in reciprocal space. Thus, XCCA provides a pathway toward reconstructing the 3D information, even without orientational alignment of the crystals.

From an experimental perspective, successful application of XCCA requires X-ray pulses shorter than the rotational diffusion timescale of the crystals, so that the structure is not moving significantly during an X-ray exposure (Mendez *et al.*, 2016 ▸). In addition, sufficient statistical sampling, *i.e.*, a large number of single-shot diffraction patterns, is essential for reliable correlation analysis and extraction of meaningful structural information (Möller *et al.*, 2025 ▸). To satisfy these requirements, Möller *et al.* employed the unique beam properties of the European X-ray free-electron laser (XFEL), including femtosecond pulse duration and a large number of pulses per second.

While some previous studies have applied femtosecond XCCA to investigate atomic-scale defects (Mendez *et al.*, 2014 ▸; Mendez *et al.*, 2016 ▸; Niozu *et al.*, 2020 ▸), these earlier works primarily focused on correlations between a limited number of scattering vectors, which restricted their ability to explore the full 3D reciprocal space. Möller and co-workers advanced this approach by analyzing the entire 3D correlation map, thereby enabling a more comprehensive assessment of the 3D reciprocal space. They further performed numerical simulations of the correlation map and demonstrated that structural properties such as stacking fault density and crystal size can be disentangled by focusing on different features in the correlation map. Based on specific peak-broadening features in the correlation map, they identified a time-dependent reduction in stacking fault density, suggesting annealing or growth of the crystals as they travel along the liquid jet.

Future applications of femtosecond XCCA will enable even more detailed analyses of disordered structures forming in crystallization processes. As Möller *et al.* mentioned, the next steps will involve probing the earliest stages of crystal nucleation and growth, as well as developing a model-free approach to reconstruct the 3D reciprocal space from the correlation maps. Such advancements will ultimately enable a more comprehensive understanding of crystallization processes in a diverse range of materials, from ice formation in the atmosphere (Lupi *et al.*, 2017 ▸) to the controlled synthesis of advanced materials.

## Figures and Tables

**Figure 1:**
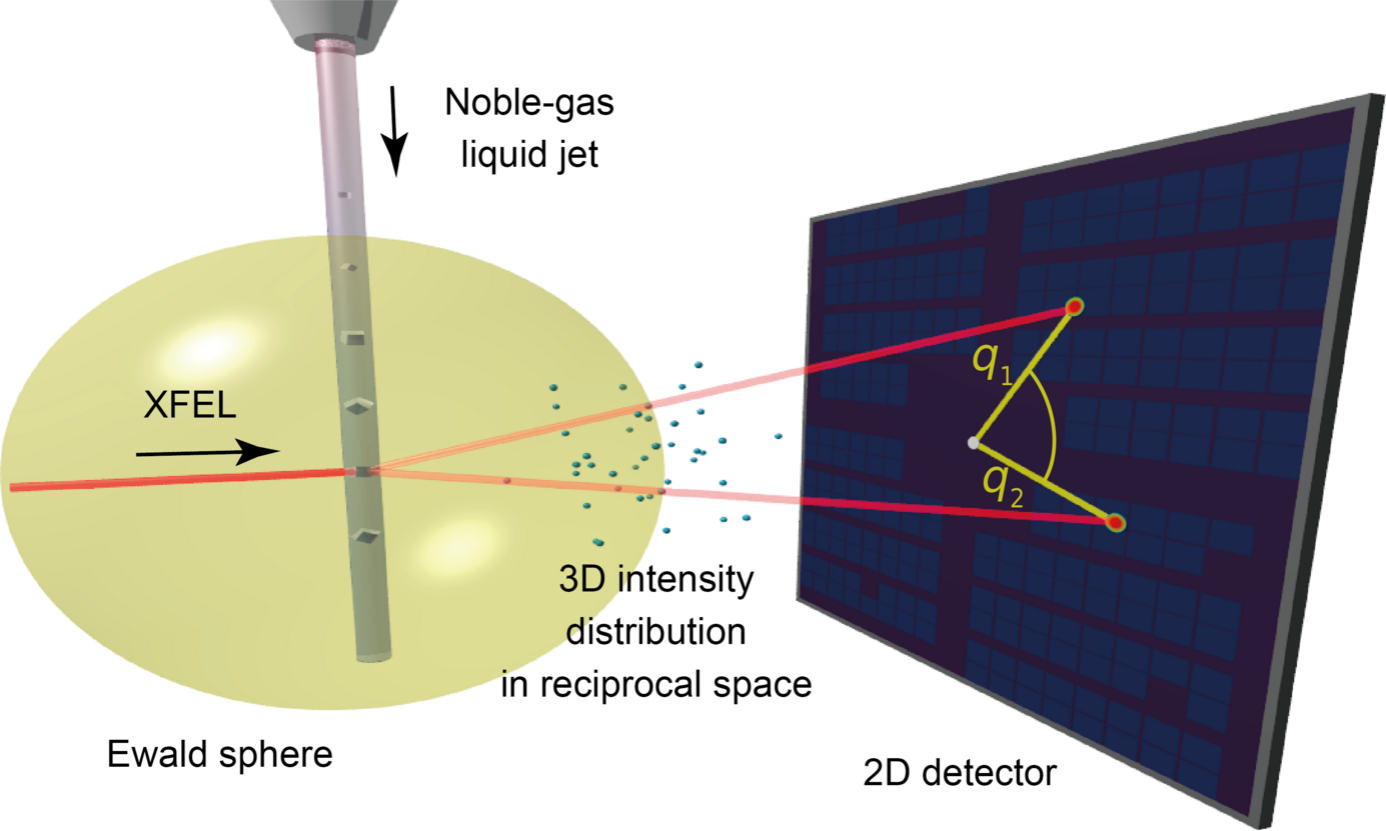
Experimental geometry and fundamental principles of XCCA (after Möller *et al.*, 2025 ▸). A single-shot diffraction pattern captures a 2D slice of the 3D intensity distribution in reciprocal space, determined by the geometry of the Ewald sphere. In XCCA, azimuthal intensity correlations of the 2D pattern are analyzed to access the 3D reciprocal space.
